# The incidence of pain and its association with quality of life in patients with peritoneal dialysis

**DOI:** 10.1080/0886022X.2022.2068444

**Published:** 2022-05-02

**Authors:** Chunyan Yi, Hongjian Ye, Jianxiong Lin, Yao Chang, Xiaodan Zhang, Ting Zhou, Rui Yang, Xiao Yang

**Affiliations:** a Department of Nephrology, The First Affiliated Hospital of Sun Yat-sen University, Guangzhou, China; b Key Laboratory of Nephrology, National Health Commission and Guangdong Province, Guangzhou, China

**Keywords:** Peritoneal dialysis, pain, quality of life

## Abstract

**Background:**

The aims of this study were to investigate the incidence of pain in peritoneal dialysis (PD) patients and to analyze the correlation between pain and quality of life.

**Methods:**

PD patients who followed up in our PD center from March 2016 to December 2017 were included. The Short-Form McGill Pain Questionnaire was used to assess pain status. Depression status, sleep quality, quality of life and clinical data were also collected.

**Results:**

A total of 463 PD patients were included, of whom 153 patients (33.1%) with pain. The main cause of pain was calcium and phosphorus metabolism disorder (51.6%). About 101 patients (66.0%) had multiple sites of pain, and 28 patients (18.3%) with pain were treated with analgesic drugs. Binary Logistic regression analysis showed that older age (OR = 1.026; *p* = 0.032) and higher intact parathyroid hormone level (OR = 1.043; *p* = 0.040) were independent risk factors for pain in PD patients. Multivariate analysis showed that score of pain rating index was an independent risk factor for depressive symptoms (OR = 1.100; *p* = 0.015), the score of Pittsburgh sleep quality index (*B* = 0.005; *p* = 0.044) and the score of physical component scale (*B*= −0.727; *p* = 0.016) in PD patients.

**Conclusions:**

The incidence of pain in PD patients was 33.1%. Older age and higher intact parathyroid hormone level were independent risk factors for pain. Pain was independently associated with depressive symptoms, sleep quality and quality of life in PD patients.

## Introduction

Pain refers to an unpleasant feeling and emotional experience that is associated with existing or potential tissue damage or is described as tissue damage [1], which is a severe and common symptom in patients receiving dialysis but remains inadequately managed in clinical practice. Patients with end stage renal disease (ESRD) may occur pain due to primary renal disease (such as renal stones, hydronephrosis, polycystic renal disease), renal failure (such as renal osteodystrophy, calcification defense), renal replacement therapy [such as abdominal distension caused by peritoneal dialysis (PD), steal away syndrome caused by hemodialysis (HD)], or other complications (such as diabetes, arthritis, nerve or vascular disease). The literature reported that the incidence of pain in HD patients was ranged from 50% to 82% [2–5]. Previous studies have shown that pain was correlated to depression [6], sleep disorders [7], quality of life (QOL) [8], and hospitalization [6] in HD patients. In addition, pain during non-dialysis period was independently correlated with death in HD patients [9]. However, very few studies investigated the incidence and the impact of pain in PD patients, and the sample sizes of these studies were small [10–12]. Therefore, this study aimed to investigate the incidence of pain in PD patients and to analyze the influence factors for pain and its impact on the QOL.

## Materials and methods

### Participants

This cross-sectional study investigated the PD patients who followed up in a single PD center of Southern China between March 2016 and December 2017. The inclusion criteria were age more than 18 years, receiving PD treatment more than 3 months and completing the questionnaire survey independently. Patients who had infection occurred in the last three months, acute cerebrovascular accident or paralysis, trauma, tumor, previous cervical and lumbar spine diseases or were unwilling to participate were excluded in this study. This study was approved by the Human Ethics Committee of Sun Yat-sen University and the written informed consent of patients was obtained.

### Measurement tools

The Short-Form McGill Pain Questionnaire (SF-MPQ) was used to assess the pain of PD patients. This questionnaire was a multidimensional measure of perceived pain in adults. The questionnaire included pain rating index (PRI) and visual analog scale (VAS). PRI was composed of 11 sensory items and 4 affective items, which were scored from 0 (no pain) to 3 (severe pain) points. The score of PRI was calculated by the scores of 15 items, which was ranged from 0 to 45, with higher scores indicating greater levels of pain. The score of VAS was range from 0 to 100 points for average pain. The validity and reliability of this questionnaire have been demonstrated in Chinese population [13].

The Beck Depression Inventory (BDI) was used to assess the psychological status of PD patients. There were 21 items in the scale, and each item was scored from 0 to 3 points. The total score was the sum of the score of each item. The reliability and validity of Chinese version was acceptable [14]. This study defined a total score of 14 or above as having depressive symptoms.

The Chinese version of Pittsburgh sleep quality index (PSQI) was used to assess the sleep quality of PD patients in the last month [15]. The PSQI was composed of seven different components including subjective sleep quality, sleep latency, habitual sleep efficiency, nighttime disturbances, sleep duration, use of sleep medications, and daytime dysfunction. Each component was scored from 0 to 3. The sum of these components generates a total score ranging from 0 to 21. The higher total score of PSQI indicated the worse sleep quality.

QOL was assessed by the medical outcomes study short form-36 (SF-36) [16]. It was a self-administered 36-item questionnaire, which was composed of eight dimensions including physical functioning, role limitation due to physical problem, bodily pain, general health, vitality, social functioning, role limitation due to emotional problem and mental health. Each dimension was scored from 0 to 100. It could also be divided into two components: the average score of the first four dimensions belong to the physical component scale (PCS), and the remaining four dimensions belong to the mental component scale (MCS). Based on the reference, total score of QOL was arithmetic averaging of the eight SF-36 domains scores [17]. The higher score of the scale indicated the better QOL. Zhao et al. [18] reported that the internal reliability of each dimension of the Chinese SF-36 scale was 0.603∼0.974.

### Data collection

At the time of PD patients being enrolled, the investigator explained the purpose and significance of the investigation to the patients, and the questionnaire was issued after obtaining the cooperation of the patients. Then the researchers checked the completeness and authenticity of the questionnaire and eliminated the invalid questionnaire. The demographic, clinical and laboratory data of patients were collected during the same period. Demographic data included age, gender, primary renal disease, diabetes mellitus and hyperuricemia before dialysis. Clinical data included duration of PD, drugs, urine output, blood pressure and body mass index. Laboratory data included hemoglobin, high-sensitivity C-reactive protein, serum albumin, serum calcium, serum phosphorus, intact parathyroid hormone, total cholesterol, triglycerides, serum sodium, serum potassium, uric acid, blood urea nitrogen, serum creatinine, residual glomerular filtration rate, and urea clearance index (Kt/V). The Charlson comorbidity index (CCI) [19] was used to assess comorbidities of PD patients.

### Statistical analysis

Continuous variables approximately normally distributed were described as mean ± standard deviation, and compared by independent sample *t* test. Skewed continuous variables were described as median and interquartile range, and compared by Mann–Whitney *U* test. Categorical variables were described as frequency and percentage, and compared by the Chi-square test. Spearman’s correlation analysis, logistic regression analysis, or linear regression analysis were used to analyze the influence factors for pain, and the relationshipbetween pain and depressive symptoms, sleep quality and quality of life. Two-sided *p* < 0.05 was considered statistically significant. All analyses were performed with SPSS version 16.0 (SPSS, Chicago, IL).

## Results

**Figure 1.**
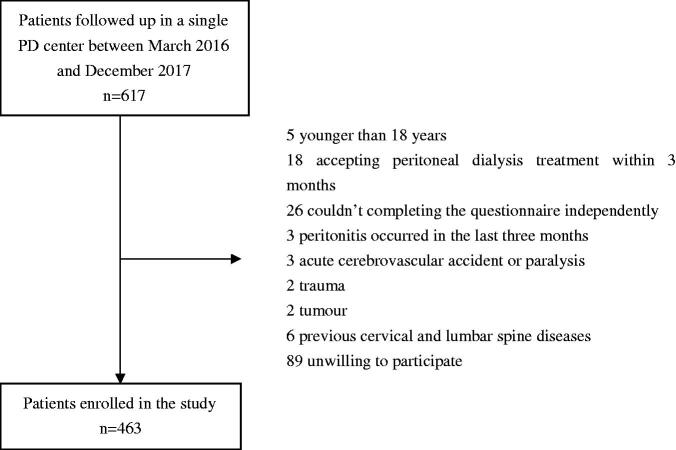
Flow chart.

A total of 463 PD patients were included in this study (Figure 1). The mean age was 48.5 ± 13.9 years, 251 (54.2%) patients were male, and 15.1% with diabetic nephropathy. The median duration of PD was 37.7 (17.5∼66.6) months. Among them, 153 (33.1%) PD patients experienced pain. In PD patients with pain, the median score of PRI was 2 (1∼5) points, and the median score of VAS with 20 (15∼50) points. The locations of pain were head (*n* = 1, 0.7%), neck (*n* = 6, 3.9%), trunk (*n* = 52, 34.0%), and limbs (*n* = 123, 80.4%). And 101 patients (66%) had multiple sites of pain, and 28 patients (18.3%) with pain were treated with analgesics. The causes of pain were calcium and phosphorus metabolism disorders (*n* = 79, 51.6%), hyperuricemia (*n* = 74, 48.4%), diabetes (*n* = 10, 6.5%), senile degenerative disease (*n* = 7, 4.6%), lower extremity arterial occlusion (*n* = 4, 2.6%), and other reasons (*n* = 30, 19.6%).

Compared with patients without pain, the patients with pain had older age, longer duration of PD, higher CCI score, body mass index, high-sensitivity C-reactive protein, serum calcium and triglycerides, and lower urine output and diastolic blood pressure (all *p* < 0.05) (Table 1). Binary logistic regression analysis showed that older age and higher level of intact parathyroid hormone were independent risk factors for pain in PD patients (all *p* < 0.05) (Table 2).

**Table 1.** Comparison of demographic data and clinical data between non-pain group and pain group.

Variables	Total (*n* = 463)	Non-pain group (*n* = 310)	Pain group (*n* = 153)	*p* values
Age (years)	48.5 ± 13.9	46.2 ± 13.5	53.2 ± 13.5	<0.001
Male (*n*, %)	251(54.2%)	176 (56.8%)	75 (49.0%)	0.115
Primary renal disease (*n*, %)				0.231
Glomerulonephritis	300 (64.8%)	210 (67.7%)	90 (58.8%)	
Diabetic nephropathy	70 (15.1%)	43 (13.9%)	27 (17.7%)	
Renal vascular diseases	39 (8.4%)	22 (7.1%)	17 (11.1%)	
Other	54 (11.7%)	35 (11.3%)	19 (12.4%)	
Diabetes mellitus (*n*, %)	93 (20.1%)	56 (18.1%)	37 (24.2%)	0.122
Hyperuricemia before dialysis (*n*, %)	42 (9.1%)	27 (8.7%)	15 (9.8%)	0.700
Charlson comorbidity index score (points)	3.0 (2.0∼4.0)	3.0 (2.0∼4.0)	4.0 (3.0∼5.0)	<0.001
Duration of peritoneal dialysis (months)	37.7 (17.5∼66.6)	34.0 (16.2∼60.3)	48.0 (23.5∼74.3)	0.002
Use of painkiller (*n*, %)	28 (6.1%)	0 (0.0%)	28 (18.3%)	<0.001
Drugs used to treat hyperuricemia (*n*, %)	69 (14.9%)	43 (13.9%)	26 (17.0%)	0.375
Drugs used to treat metabolic disorders of bone minerals (*n*, %)	326 (70.4%)	214 (69.0%)	112 (73.2%)	0.355
Urine output (ml/d)	300.0 (0.0∼775.0)	337.5 (10.0∼842.5)	150.0 (0.0∼550.0)	0.006
Systolic blood pressure (mmHg)	135.6 ± 21.9	135.9 ± 20.9	135.0 ± 23.9	0.702
Diastolic blood pressure (mmHg)	83.1 ± 13.3	84.6 ± 12.6	80.1± 14.1	0.001
Body mass index (kg/m^2^)	22.2 ± 3.2	22.0 ± 3.2	22.6 ± 3.2	0.048
Hemoglobin (g/L)	113.4 ± 19.5	114.2± 20.1	111.9 ± 18.4	0.247
Serum albumin (g/L)	37.0± 4.1	37.3 ± 4.0	36.6 ± 4.2	0.079
High-sensitivity C-reactive protein (mg/L)	1.6 (0.6∼5.0)	1.3(0.6∼3.8)	2.3 (0.9∼8.4)	<0.001
Serum calcium (mmol/L)	2.3 ± 0.2	2.2 ± 0.2	2.3 ± 0.2	0.017
Serum phosphorus (mmol/L)	1.6 (1.3∼1.9)	1.6 (1.3∼1.9)	1.7 (1.3∼2.1)	0.086
Intact parathyroid hormone (pg/ml)	329.3 (158.1∼647.8)	331.5 (165.0∼569.7)	329.1 (146.1∼718.2)	0.451
Total cholesterol (mmol/L)	4.9 (4.2∼5.7)	4.9 (4.2∼5.7)	4.8 (4.1∼5.6)	0.522
Triglycerides (mmol/L)	1.5 (1.1∼2.2)	1.4 (1.0∼2.1)	1.6 (1.2∼2.8)	0.002
Blood urea nitrogen (mmol/L)	16.8 (14.4∼20.4)	16.8 (14.1∼20.4)	14.7 (16.9∼20.4)	0.838
Serum creatinine (µmol/L)	1005.0 (802.0∼1227.0)	1020.0 (814.0∼1257.5)	973.0 (776.0∼1164.0)	0.078
Serum sodium (mmol/L)	138.3 ± 3.9	138.5 ± 4.2	138.0 ± 3.1	0.179
Serum potassium (mmol/L)	4.1 ± 0.7	4.1 ± 0.7	4.1 ± 0.7	0.326
Uric acid (µmol/L)	404.4± 73.0	402.0 ± 71.0	409.4 ± 77.0	0.305
Residual renal function (ml/min/1.73 m^2^)	0.9 (0.0∼2.9)	1.1 (0.1∼2.9)	0.5 (0.0∼2.7)	0.124
Clearance index of urea	2.1 (1.8∼2.4)	2.1 (1.8∼2.4)	2.1 (1.8∼2.4)	0.810
Total score of Beck Depression Inventory (points)	10.0 (5.0∼16.0)	9.0 (4.0∼16.0)	11.0 (5.5∼18.5)	0.024
Total score of Pittsburgh Sleep Quality Index (points)	7.0 (5.0∼11.0)	7.0 (4.0∼11.0)	8.5 (5.0∼13.0)	0.005
Total score of quality of life (points)	60.0 ± 17.8	60.7 ± 17.3	58.5 ± 18.8	0.223
Physical component scale (points)	56.9± 18.4	58.1 ± 17.4	54.6 ± 20.1	0.055
Mental component scale (points)	63.0 ± 19.7	63.3± 19.7	62.5 ± 19.9	0.681

**Table 2.** The influence factors for pain in peritoneal dialysis patients.

Variables	Univariate logistic regression analysis			Multiple logistic regression analysis		
	OR	95%CI	*p* values	OR	95%CI	*p* values
Age (per 1 year)	1.038	1.023∼1.054	<0.001	1.026	1.002∼1.051	0.032
Male (yes)	0.732	0.496∼1.080	0.116	0.786	0.513∼1.204	0.268
Duration of peritoneal dialysis (per 1 month)	1.010	1.004∼1.016	0.001	1.005	0.997∼1.012	0.198
Charlson comorbidity index (per 1 point)	1.235	1.114∼1.369	<0.001	1.044	0.879∼1.241	0.620
Urine output (per 1 ml/d)	0.999	0.999∼1.000	0.007	1.000	0.999∼1.000	0.560
Diastolic blood pressure (per 1 mmHg)	0.974	0.959∼0.989	0.001	0.992	0.975∼1.010	0.374
Body mass index (per 1 kg/m^2^)	1.062	1.000∼1.128	0.049	1.021	0.953∼1.093	0.557
High-sensitivity C-reactive protein (per 1 mg/L)	1.038	1.013∼1.064	0.003	1.017	0.991∼1.044	0.198
Serum calcium (per 1 mmol/L)	3.441	1.227∼9.645	0.019	2.640	0.862∼8.085	0.089
Intact parathyroid hormone (per 100 pg/ml)	1.051	1.015∼1.089	0.005	1.043	1.002∼1.086	0.040
Triglycerides (per 1 mmol/L)	1.153	1.011∼1.314	0.034	1.056	0.915∼1.219	0.454

OR: odds ratio; 95% CI: 95% confidence interval.

The patients with pain had higher BDI score and PSQI scorecompared with those patients without pain (all *p*＜0.05) (Table 1). Spearman’s correlation analysis showed that the score of PRI was positively correlated with BDI score (*r*’=0.133; *p* = 0.004) and PSQI score (*r*’=0.162; *p* = 0.001), and negatively correlated with PCS score (*r*’= −0.091; *p* = 0.049). No significant correlation was found between the score of PRI and total score of QOL (*r*’= −0.060; *p* = 0.194) or the score of MCS (*r*’= −0.032; *p* = 0.493). Binary Logistic regression analysis showed that the score of PRI was an independent risk factor for depression symptoms in PD patients (*p* = 0.015) (Table 3). Multiple linear regression analysis showed that the score of PRI was an independent influence factor for the score of PSQI (*p* = 0.044) and the score of PCS (*p* = 0.016) in PD patients after adjustment for other confounders (Tables 4 and 5).

**Table 3.** The impact of pain rating index score on depression symptoms of peritoneal dialysis patients.

Variables	Univariate logistic regression analysis			Multiple logistic regression Analysis		
	OR	95%CI	*p* Values	OR	95%CI	*p* Values
Age (per 1 year)	0.996	0.983∼1.010	0.603	0.976	0.954∼0.999	0.041
Male (yes)	0.961	0.658∼1.403	0.836	1.007	0.639∼1.587	0.976
Duration of peritoneal dialysis (per 1 month)	1.013	1.007∼1.019	<0.001	1.008	1.001∼1.015	0.033
Charlson comorbidity index (per 1 point)	1.002	0.905∼1.108	0.974	1.063	0.895∼1.262	0.489
Urine output (per 1 ml/d)	0.999	0.999∼0.999	<0.001	0.999	0.999∼1.000	0.025
High-sensitivity C-reactive protein (per 1 mg/L)	1.025	1.002∼1.049	0.037	1.013	0.988∼1.038	0.318
Serum phosphorus (per 1 mmol/L)	2.029	1.340∼3.073	0.001	1.633	0.972∼2.746	0.064
Serum creatinine (per 1 µmol/L)	1.001	1.000∼1.002	0.005	1.000	0.999∼1.001	0.885
Total score of pain rating index (per 1 point)	1.129	1.050∼1.214	0.001	1.100	1.019∼1.188	0.015

OR: odds ratio; 95% CI: 95% confidence interval.

**Table 4.** The impact of score of pain rating index on total score of Pittsburgh sleep quality index of peritoneal dialysis patients.

Variables	Unitary linear regression analysis				Multiple linear regression analysis			
	*B*	Beta	*t*	*p* values	*B*	Beta	*t*	*p* values
Age (per 1 year)	0.002	0.230	4.927	<0.001	0.003	0.283	3.968	<0.001
Male (yes)	−0.028	−0.098	−2.057	0.040	−0.190	−0.067	−1.454	0.147
Duration of peritoneal dialysis (per 1 month)	0.001	0.187	3.959	<0.001	0.0003	0.064	1.250	0.212
Charlson comorbidity index (per 1 point)	0.008	0.103	2.162	0.031	−0.012	−0.162	−2.234	0.026
Urine output (per 1 ml/d)	−0.00005	−0.203	−4.321	<0.001	−0.00002	−0.092	−1.747	0.081
Diastolic blood pressure (per 1 mmHg)	−0.002	−0.153	−3.227	0.001	−0.001	−0.064	−1.281	0.201
High-sensitivity C-reactive protein (per 1 mg/L)	0.002	0.096	2.020	0.044	0.00008	0.005	0.100	0.920
Serum phosphorus (per 1 mmol/L)	0.045	0.151	3.176	0.002	0.037	0.123	2.582	0.010
Total score of pain rating index (per 1 point)	0.008	0.180	3.826	<0.001	0.005	0.099	2.023	0.044

**Table 5.** The impact of pain rating index score on physical component scale of peritoneal dialysis patients.

Variables	Unitary linear regression analysis				Multiple linear regression analysis			
	*B*	Beta	*t*	*p* values	*B*	Beta	*t*	*p* values
Age (per 1 year)	−0.152	−0.114	−2.469	0.014	0.063	0.047	0.649	0.517
Male (yes)	−1.061	−0.029	−0.618	0.537	−0.266	−0.007	−0.156	0.876
Duration of peritoneal dialysis (per 1 month)	−0.062	−0.108	−2.339	0.020	−0.019	−0.032	−0.626	0.532
Charlson comorbidity index (per 1 point)	−1.514	−0.153	−3.328	0.001	−1.294	−0.131	−1.822	0.069
Urine output (per 1 ml/d)	0.005	0.158	3.427	0.001	0.003	0.103	1.993	0.047
Diastolic blood pressure (per 1 mmHg)	0.200	0.144	3.133	0.002	0.127	0.092	1.847	0.065
Serum albumin (per 1 g/L)	0.536	0.120	2.585	0.010	0.055	0.012	0.238	0.812
Triglycerides (per 1 mmol/L)	1.248	0.099	2.131	0.034	1.920	0.152	3.127	0.002
Total score of pain rating index (per 1 point)	−0.927	−0.150	−3.265	0.001	−0.727	−0.118	−2.427	0.016

## Discussion

In this cross-sectional study, it was found that 33.1% of PD patients occurred pain symptoms. Older age and higher level of intact parathyroid hormone were independent risk factors for pain in PD patients. The score of PRI was an independent influence factor for depression symptoms, the score of PSQI and the score of PCS in PD patients.

Pain was one of the common symptoms in patients with ESRD. Currently, few data on the management of pain were available in PD patients. This study found that the incidence of pain in PD patients was 33.1%, which was lower than that of HD patients (50–82%) [2–5]. During the dialysis process, HD patients might occur pain due to needle insertion or muscle cramps, abdominal or cardiac pain due to intradialytic ischemia, or headaches [20]. However, PD patients rarely had such experience. This might be the reason for the different incidence of pain between PD and HD patients. Similar to HD patients [4,20,21], the results of this study showed that two-thirds of PD patients had multiple sites of pain, but only 18.3% of pain patients had used analgesics. It was suggested that the pain problem of PD patients deserved further attention and management by clinical medical staff.

This study showed that the disorder of calcium and phosphorus metabolism was the main cause of pain in PD patients, and higher intact parathyroid hormone was one of independent risk factors for pain in PD patients. Elsurer et al. [22] also found that intact parathyroid hormone was an independent factor in patients with bone pain in HD patients. More attention should be pay on the regular detection of bone metabolism-related indicators for PD patients, adjustment of medication and peritoneal dialysis regimens, timely correction of calcium and phosphorus metabolism disorders, and reduction of the incidence of renal bone disease. It was reported that high symptom burden was prevalent in older ESRD patients [23]. This study also found that older age was an independent risk factor for pain in PD patients. In HD patients, few literatures reported the correlation between age and pain [24], while other literatures did not found this correlation [25]. As patients get older, their perception of pain may be influenced by many factors. Complications such as osteoarthritis, chronic low back pain, rheumatoid arthritis, and polymyalgia rheumatica increase the rate of pain. Increased pain thresholds or psychological problems such as depression and stress make the perception of pain decreased. It was suggested that further research was needed to determine the role of age in the development of pain in dialysis patients.

A Canadian study showed that pain was independently related to depression and sleep disorders in HD patients [7]. Elsurer et al. [22] investigated 95 HD patients and found that the intensity of chronic bone pain was negatively correlated with the PCS and MCS of SF-36. Davison et al. [25] longitudinally observed the relationship between pain and symptom burden and QOL changes in 591 HD patients, and the results showed that pain was independently related to changes in physical health and mental health. Belayev et al. [26] also found that pain was independently related to the decline in QOL in HD patients. Harris et al. [9] investigated the potential relationship between pain, sleep, QOL and survival in HD patients, and found that the results reported by patients might be an important tool that affected the QOL and survival of ESRD patients. The results of this study also found that the score of PRI was an independent influence factor for depression symptoms, the score of PSQI and the score of PCS in PD patients. The above analysis suggested that pain might affect negative emotions, sleep quality, and QOL of dialysis patients.

A limitation of this study was that all participants were from a single PD center of Southern China. The results of this study might not be applicable to all PD patients. Another limitation was cross-sectional design of this study, which could not examine the causal relationship between pain and the potential risk factors.

In conclusion, this study demonstrated that the incidence of pain in PD patients was 33.1%. Older age and higher level of intact parathyroid hormone were independent risk factors for pain. Pain was independently related to depression symptom, sleep quality and QOL in PD patients. Understanding experiences of pain in PD patients could inform strategies to address this symptom. It was suggested that pain problem of PD patients deserved more attention and a strong imperative to establish chronic pain management of PD patients as a clinical and research priority.

## Data Availability

The datasets generated and/or analyzed during the current study are available from the corresponding author on reasonable request.

## Abbreviations

BDI: Beck Depression Inventory
CCI: Charlson comorbidity index
ESRD: end-stage renal disease
HD: hemodialysis
Kt/V: urea clearance index
MCS: mental component scale
PCS: physical component scale
PD: peritoneal dialysis
PRI: pain rating index
PSQI: Pittsburgh sleep quality index
QOL: quality of life
SF-36: medical outcomes study short form-36
SF-MPQ: Short-Form McGill Pain Questionnaire
VAS: visual analog scale
